# Principal component analysis of cytokine signature in COVID-19 and Long COVID

**DOI:** 10.3389/fimmu.2026.1717107

**Published:** 2026-02-26

**Authors:** Zoia R. Korobova, Areg A. Totolian

**Affiliations:** 1 Laboratory of Molecular Immunology, Saint Petersburg Pasteur Institute, Saint Petersburg, Russia; 2 Department of Immunology, Faculty of Medicine, First Pavlov State Medical University of St. Petersburg, Saint Petersburg, Russia

**Keywords:** long COVID, post-COVID condition, COVID-19, cytokines, principal component analysis, clusterization, cytokine network

## Abstract

**Introduction:**

Despite the activity of the COVID-19 pandemic being lower in the recent years, COVID-associated threat, long COVID (LC). Its clinical presentation includes nearly 200 symptoms affecting cardiovascular, respiratory, nervous systems, endocrine organs, urinary tract, and gastrointestinal systems. Cytokines serve as important biomarkers for assessing the level of immune system involvement and dysregulation in LC. Most studies on cytokine network and cytokine interactions usually address more traditional methods of statistical analysis with comparison criteria, discriminant analysis, regression. But multiplex cytokine analysis includes dozens of parameters, and requires complex assessment of the network as a whole.

**Methods:**

We analyzed data of cytokine multiplex analysis of 289 patients with COVID-19, 44 patients with LC and 51 healthy donors. PCA we identified cyotkines with the highest importance rate, and further investigated between them with the use of 3D mapping.

**Results:**

Three key clusters were identified: cluster A - IL-13, CCL7/MCP-3, IL-4; cluster B - IL-18, CCL2/MCP-1, CCL4/MIP-1β, CXCL8/IL-8, M-CSF, and cluster C - sCD40L, CXCL1/GROα, PDGF-AA, EGF, FGF-2, FLT-3L, IL-7, IL-17F.

**Discussion:**

The coordinated interactions within these clusters reveal a complex immunopathology behind LC: clsuter A repsresenting local immune responses, cluster B for neuroinflammatory processes, and cluster C for changes in the blood vessels. The results, however, leave an opening for further investigatoin and interpretation.

## 1 Introduction

Despite the activity of the COVID-19 pandemic being lower in the recent years, humanity still faces its consequences through both economic and medical points of view. One of the major COVID-associated threats is the new disease, a so-called long COVID (LC) or long-haul COVID (1). So far, this disease demonstrated high heterogeneity of symptoms combined with concerning epidemiology: nearly 6% of COVID-19 survivors (i.e., millions globally) develop LC (2).

Its clinical presentation includes nearly 200 symptoms affecting cardiovascular, respiratory, nervous systems, endocrine organs, urinary tract, and gastrointestinal systems. In 15% of cases, these symptoms persist for a long time and lead to the decline in quality of life (2). Most common symptoms, however, are usually fatigue (in over 80% of patients), cognitive dysfunction and brain fog (50-60%), and severe anxiety and/or depression (30-40%) (3). These symptoms highlight the importance of precise attention toward vulnerable society groups, e.g., fertile women, patients with autoimmune diseases, survivors of critical COVID-19, elderly, patients with endocrine disorders and those without access to medial services (4).

Key unresolved issue for LC diagnostics is the need for proper standardization. Current diagnostic criteria are mainly clinical, which leads not only to the gaping hole in laboratory markers, but also to failure to understand key aspects of disease development. In the absence of proper targets for therapy, medical professionals are limited in terms of treatment strategies for LC patients.

While the clinical manifestations of LC are increasingly well-documented, the role of SARS-CoV-2 genetic variants in influencing the risk and phenotype of long-term consequences remains an active area of investigation. Emerging evidence suggests that certain viral lineages may cause prolonged symptoms, though host factors—including genetic predisposition—likely interact with viral characteristics to determine individual outcomes. Studies have begun to explore whether specific SARS-CoV-2 variants (e.g., Delta vs. Omicron) bear different risks for developing LC (5). The continued circulation of new SARS-CoV-2 variants such as Flirt (2025), despite their potentially lower virulence, requires assessment of their potential to cause LC, particularly in immunocompromised individuals.

All of this creates a significant socioeconomic burden, characterized by high costs for long-term treatment and rehabilitation, and the need to reorganize healthcare systems to establish multidisciplinary support services. Therefore, even as we move further away from the peak of the pandemic, it remains critically important for modern medicine and healthcare to comprehensively study the pathogenesis, improve diagnostics (including the search for reliable biomarkers), develop personalized treatment and rehabilitation approaches for patients with LC, and assess the long-term effects of new viral variants and subvariants.

Cytokines serve as important biomarkers for assessing the level of immune system involvement and dysregulation in LC. These signaling molecules of polypeptide nature play a key role in organizing and coordinating immune responses: they initiate and regulate inflammation, and control processes of cell proliferation, differentiation, and apoptosis. Measuring the cytokine profile in the blood of patients with various conditions, including LC, is a highly informative method that reflects systemic immunopathological processes. In LC, changes in the levels of key pro-inflammatory (such as IL-6, TNF-α, IL-1β, IFN-γ) and anti-inflammatory (e.g., IL-10) cytokines can indicate persistent subclinical inflammation, autoimmune mechanisms, or a dysfunction in immune tolerance that underlies the syndrome’s diverse symptoms (6).

Principal component analysis (PCA) is a valuable biostatistical tool that reduces data dimensionality by creating new variables (principal components) that capture the maximum variance in the original data. PCA in immunology studies provides better insights for multi-parameter data — such as cytokine levels, cell counts, and their interactions. This method allows to highlight hidden patterns of immune activation while maximizing and reorganizing information on data variation. It also transforms multi-parameter data into low-dimension visualization.

Most studies on cytokine network and cytokine interactions usually addresses more traditional methods of statistical analysis with comparison criteria (e.g., Mann-Whitney and Kruskall-Wallis) (7), discriminant analysis (8), regression (9). But multiplex cytokine analysis includes dozens of parameters, and requires complex assessment of the network as a whole. For effective assessment of such datasets PCA works perfectly, but is rarely used (10, 11).

The goal of this study was to perform PCA to study COVID-associated changes in cytokine network, based on our previous data on acute-stage COVID-19 patients and our relatively new data on LC patients.

## 2 Methods

### 2.1 Patients’ demographics

A total of 289 samples were obtained from patients with PCR-verified COVID-19 who were hospitalized with an official COVID-19 diagnosis at two medical institutions in Saint Petersburg, Russia: the COVID-19-specialized hospital at Pavlov First Saint Petersburg State Medical University and the North-Western Scientific and Clinical Center named after L.G. Sokolov. The study cohort comprised individuals infected with different SARS-CoV-2 variants: original Wuhan strain (n = 59), variants Alpha (n = 95), Delta (n = 78), and Omicron (n = 57). Median ages (with interquartile ranges Q25–Q75) for each group were as follows: Wuhan: 58 (32–75), Alpha: 72 (28–84), Delta: 71 (45–93), Omicron: 69 (32–91). Original data on the patients’ cohort of acute COVID-19 patients is presented in (12, 13).

A separate LC group (n = 44) was defined according to WHO criteria for post-COVID condition, comprising individuals with persistent psychoneurological symptoms—such as cognitive impairment (assessed via the Montreal Cognitive Assessment) and anxiety/depression (evaluated using the Hospital Anxiety and Depression Scale)—along with somatic complaints including fatigue and dyspnea lasting more than 12 weeks after the acute infection. All LC participants had PCR-confirmed SARS-CoV-2 infection within 120–365 days prior to sample collection, were aged 18–60 years (median 38, IQR 29–48), and were predominantly female (72.7%, n = 32). All LC participants had PCR-confirmed prior SARS-CoV-2 infection, were aged 18–60 years (median 38, IQR 29–48), and were predominantly female (72.7%, n = 32). We acknowledge this gender imbalance as a limitation for the interpretation and generalizability of the results, particularly regarding male populations. The majority experienced mild acute COVID-19 (84.1%, n = 37), while 15.9% (n = 7) had moderate to severe disease. Recurrent infections were reported in 79.5% (n = 35) of participants, and only 40.9% (n = 18) had been vaccinated against COVID-19 prior to infection. Frequently reported symptoms included blood pressure fluctuations (100%), hair loss (56.8%), poor appetite (52.3%), and nonspecific abdominal pain (45.5%). Comorbidities in remission were present in 75% (n = 33) of participants, including gastrointestinal (31.8%), renal/urinary (18.2%), and cardiovascular disorders (6.8%); 22.7% (n = 10) had a body mass index exceeding 25 kg/m^2^. Original data on LC patients is presented in (14).

Additionally, plasma samples were included from 51 healthy donors with no history of COVID-19, collected during the initial phase of the pandemic (January to March 2020). Median age for healthy donors comprised 49 (24–69).

The interpretation of our results should consider certain limitations. The sample size of the LC cohort, while carefully characterized, was constrained by recruitment realities. A *post-hoc* Cohen d assessment of statistical power revealed a spectrum of effect sizes across the dysregulated cytokine network among studied cohorts. The findings related to cytokines with smaller effect sizes should be considered preliminary and require validation in larger, independent cohorts. We did not adjust for confounding factors like age, sex, or comorbidities, which differed between variant groups and the Long COVID cohort. These demographic and clinical differences may have influenced the cytokine profiles independently of the viral strain or post-acute condition. Our findings should therefore be interpreted as associations, and future studies adjusting for these covariates are needed.

### 2.2 Blood collection and plasma isolation

Peripheral blood (5 mL) was collected in K3 EDTA tubes (VACUETTE^®^, Griener Bio-One, Kremsmünster, Austria) prior to therapeutic interventions. Plasma was isolated via centrifugation at 300g for 7 min at 25 °C. Aliquots were stored at −80 °C until cytokine analysis.

### 2.3 Cytokine assessment

A panel of 47 immune mediators (cytokines, chemokines, growth factors) was quantified using the MILLIPLEX^®^ MAP Human Cytokine/Chemokine Magnetic Bead Panel (MilliporeSigma, Cat. # HCYTOMAG-60K, MA, USA) on the Luminex^®^ MAGPIX™ (Luminex, TX, USA) platform. Analytes included interleukins and selected pro-inflammatory cytokines (IL-1α, IL-1β, IL-2, IL-3, IL-4, IL-5, IL-6, IL-7, IL-9, IL-12 (p40), IL-12 (p70), IL-13, IL-15, IL-17A/CTLA8, IL-17-E/IL-25, IL-17F, IL-18, IL-22, IL-27, IFNα2, IFNγ, TNFα, TNFβ/Lymphotoxin-α[LTA]); chemokines (CCL2/MCP-1, CCL3/MIP-1α, CCL4/MIP-1β, CCL7/MCP-3, CCL11/Eotaxin, CCL22/MDC, CXCL1/GROα, CXCL8/IL-8, CXCL9/MIG, CXCL10/IP-10, CX3CL1/Fractalkine); anti-inflammatory cytokines (IL-1Ra, IL-10); growth factors (EGF, FGF-2/FGF-basic, Flt-3 Ligand, G-CSF, M-CSF, GM-CSF, PDGFAA, PDGFAB/BB, TGFα, VEGF-A); and sCD40L. Samples were processed per manufacturer protocol: 25 μL plasma was incubated with antibody-conjugated beads overnight (4 °C), followed by detection with biotinylated secondary antibodies and streptavidin-PE.

### 2.4 Statistical analysis and PCA

The following R packages (version 4.3.1) were used in the analysis: tidyverse (1.3.2) for data preprocessing, FactoMineR (2.8) for performing PCA, ggplot2 (3.4.2) for plotting, and flextable (0.9.0) for generating tables, effsize (0.8.1) for *post-hoc* power calculation.

Raw intensity values underwent sequential processing: conversion to numeric format, base−10 logarithmic transformation with a pseudocount [log10(x + 1)] to normalize the distribution and handle zeros, and removal of the few rows containing missing values (constituting <1% of all data points). Given the minimal and random nature of the missing data, imputation was deemed unnecessary, and a complete-case analysis was employed to preserve data integrity without introducing imputation-related assumptions.

**Figure 1:**
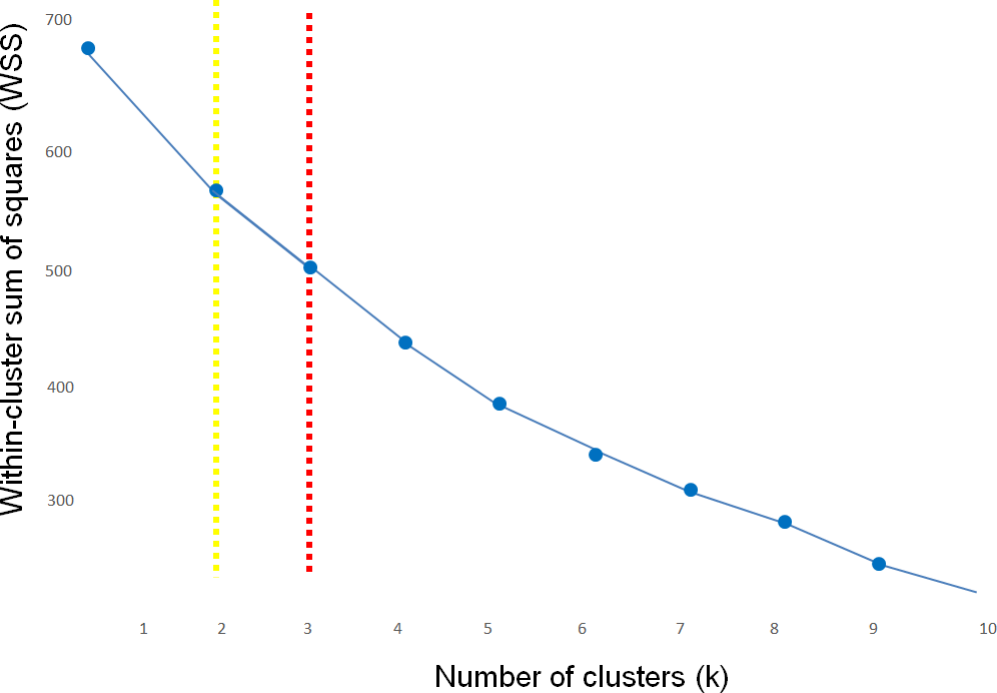
Determination of the optimal cluster number (k) for k-means analysis using the elbow method. The plot displays the within-cluster sum of squares (WSS) on the Y-axis against the number of clusters (k) on the X-axis. The point of inflection (elbow), where the rate of WSS decrease sharply slows, was identified at k=2 (indicated by the yellow dotted line) by the standard algorithm. However, based on the 3D spatial distribution of the data and the requirement for biological interpretability, a k=3 solution (indicated by the red dotted line) was selected for the final analysis, as it provided a more meaningful resolution of functionally distinct cytokine clusters.

For principal component analysis (PCA), the data were scaled to unit variance and zero mean using the scale() function, followed by singular value decomposition using the FactoMineR package. The importance of biomarkers was quantified by the length of their loading vector (Importance = √(PC1^2^ + PC2^2^)). Based on these calculations, a ranked table of cytokines was generated. The plot used for visualization was a heatmap created via ggplot2. We considered Importance value high at >0.7. For clusterization, the optimal number of k-means clusters was calculated by the elbow method (**Figure 1**). While the elbow plot suggested a potential solution at k=2, a k=3 solution was ultimately selected as it provided better biological interpretability, clearly resolving three functionally distinct cytokine clusters that aligned with known immunological pathways in LC.

To statistically validate the identified principal components and distinguish true signal from noise, we performed a permutation test with 100 iterations. This analysis confirmed that the first five principal components (PC1-PC5) were highly significant (p < 0.001), as their eigenvalues substantially exceeded those obtained from randomly permuted data. Components beyond PC5 were not significant (p = 1), indicating they likely represent random noise (**Table 1**).

**Table 1:** The first 10 principle components significance via permutation test (100 permutations).

PC	Eigenvalue	p-value	Significance
1	14.7827553	< 0.001	TRUE
2	7.2576831	< 0.001	TRUE
3	4.6492475	< 0.001	TRUE
4	2.3432898	< 0.001	TRUE
5	1.9373102	< 0.001	TRUE
6	1.4329853	1	FALSE
7	1.0873611	1	FALSE
8	1.0432270	1	FALSE
9	0.9359654	1	FALSE
10	0.8461305	1	FALSE

The five significant components collectively explained 69.9% of the total variance in the dataset. Sensitivity analysis across cohorts demonstrated consistent results, with total variance explained ranging from 62.7% to 83.9% across different SARS-CoV-2 variants and LC cohort (**Table 2**).

**Table 2:** PCA sensitivity analysis results between cohorts included in the study.

Group	Total variance	Mean variance	PC1_Variance	Valid components
Wuhan	67.5	13.5	24.1	5	
Alpha	72.9	14.3	23.2	5	
Delta	83.9	16.8	41.8	5	
Omicron	62.7	12.5	19.6	5	
LC	68.8	13.8	27.0	5	

3D plot was built with the use of plotly (v. 2.5.1) and cluster (v. 2.1.8.1) packages for RStudio. For visualization we also used open access software Bioicons (https://bioicons.com/, accessed 25^th^ September, 2025) and Photopea (https://www.photopea.com/, accessed 25^th^ September, 2025).

## 3 Results

**Figure 2:**
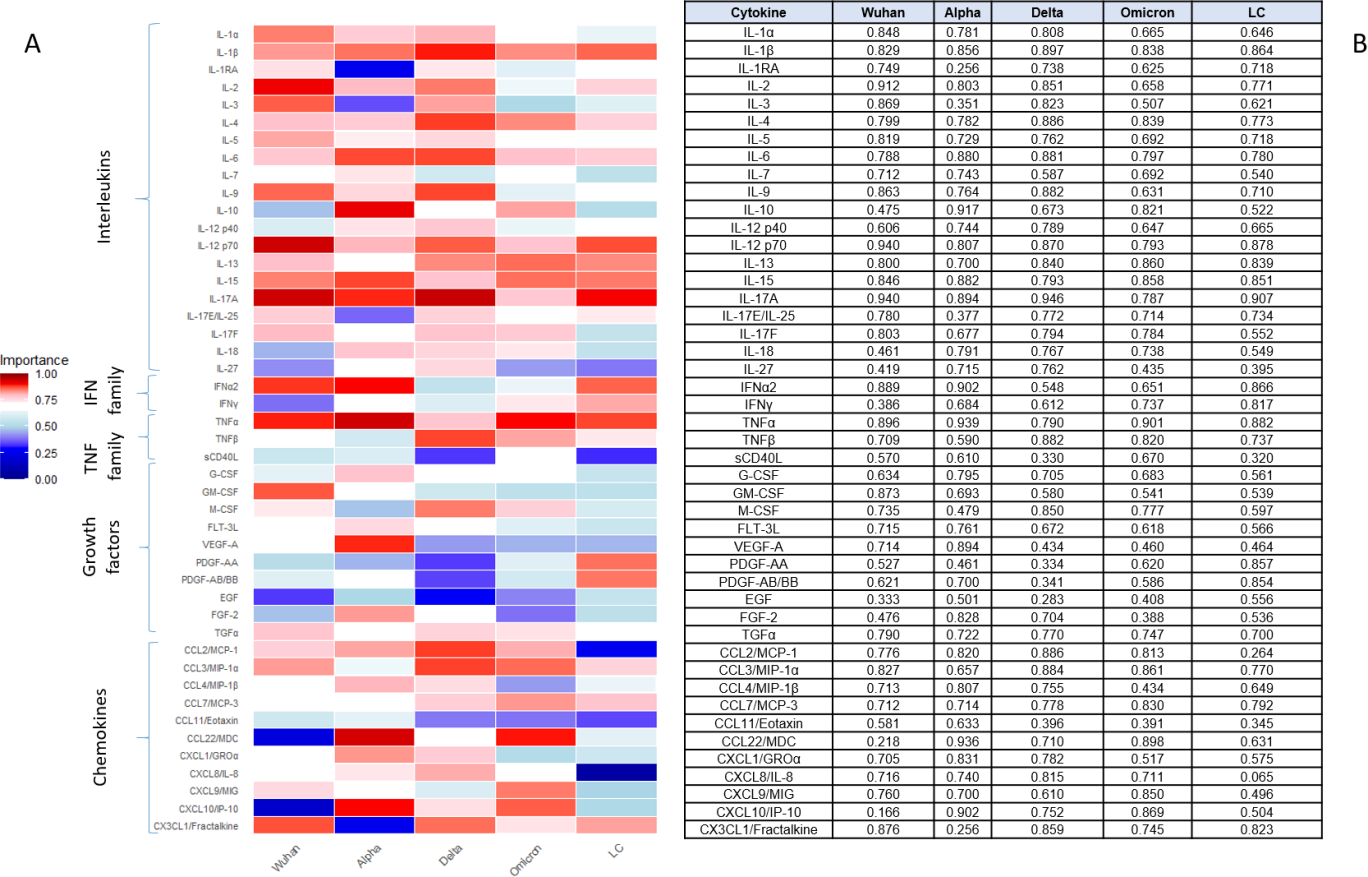
Heatmap **(A)** and table **(B)** representing results of principal component analysis and calculation of Importance value (length of loading vector). Studied cohorts included patients infected with original SARS-CoV-2 strain (Wuhan, *n=*59), genetic variants Alpha (*n=*95), Delta (*n=*78) and Omicron (*n=*69), and patients with Long COVID (LC, *n=*44).

As the first step of this study, we calculated Importance [√(PC1^2^ + PC2^2^)] value for each cytokine for all 4 genetic variants and LC (**Figure 2**).

Based on the principal component analysis, several cytokines demonstrated high importance across multiple SARS-CoV-2 variants and in LC LC, as quantified by their loading vector magnitudes. Notably, IL-12 p70, IL-17A, and TNFα consistently exhibited high importance scores (≥0.787) in all variant groups and LC. Other cytokines, such as IL-1β, IL-15, and IL-13, also showed strong contributions to component loading in most groups. In contrast, certain mediators including EGF, CCL11/Eotaxin, and sCD40L displayed generally lower importance scores across cohorts.

As many cytokines exhibited high importance values in PCA, we chose to create loading plots specifically for those cytokines that showed statistically significant differences compared to healthy donors. We revisited our earlier data on cytokines in the studied cohorts and healthy donors, and specifically chose cytokines that demonstrated statistically significant differences at *p*>0.05 (**Table 3**).

**Table 3:** Cytokines and other molecules that demonstrated statistically significant differences to healthy donors, when compared with Mann-Whitney test with multiple comparisons correction.

Cohort	Increase in blood plasma (↑) when compared to healthy donors	Decrease in blood plasma (↓) when compared to healthy donors
Original Wuhan strain (*n=*59)	IL-1α, IL-1β, IL-1RA, IL-3, IL-5, IL-6, IL-7, IL-10, IL-12 (p70), IL-15, IL-18, IL-27, IFNα2, IFNγ, TNFα, TNFβ, G-CSF, GM-CSF, VEGF-A, PDGF-AA, PDGF-AB/BB, EGF, FGF-2, CCL2/MCP-1, CCL3/MIP-1α, CCL4/MIP-1β, CCL7/MCP-3, CXCL1/GROα, CXCL8/IL-8, CXCL9/MIG, CXCL10/IP-10, CX3CL1/Fractalkine	CCL22/MDC
Alpha variant (*n=*95)	IL-1RA, IL-6, IL-10, IL-15, IL-18, IL-27, IFNα2, IFNγ, TNFα, G-CSF, M-CSF, FLT-3L, FGF-2, CCL2/MCP-1, CCL3/MIP-1α, CCL4/MIP-1β, CCL7/MCP-3, CXCL1/GROα, CXCL8/IL-8, CXCL10/IP-10, CX3CL1/Fractalkine	CCL22/MDC
Delta variant (*n=*78)	IL-1RA, IL-6, IL-10, IL-18, IL-27, CCL2/MCP-1, CCL11/Eotaxin, CXCL8/IL-8, CXCL9/MIG, CXCL10/IP-10	CCL22/MDC
Omicron variant (*n=*69)	IL-6, IL-10, IL-18, IL-27, CCL2/MCP-1, CCL4/MIP-1β, CXCL8/IL-8, CXCL9/MIG, CXCL10/IP-10	CCL22/MDC
LC (*n=*44)	sCD40L, CXCL1/GROα, IL-13, IL-18, CCL2/MCP-1, CCL7/MCP-3, CCL4/MIP-1β, PDGF-AA	EGF, FGF-2, FLT-3L, IL-4, IL-7, CXCL8/IL-8, IL-17F, IL-22, M-CSF

**Figure 3:**
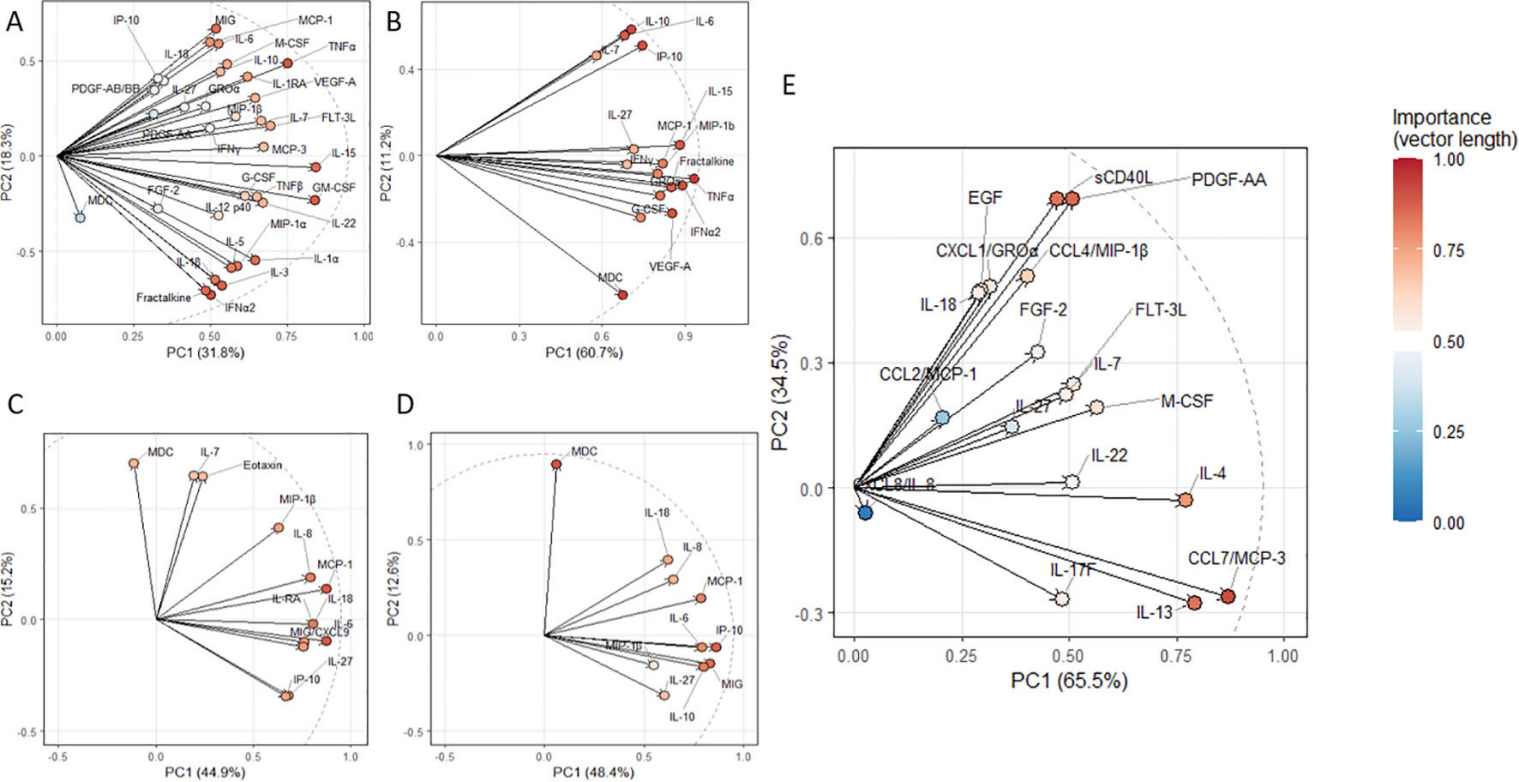
PCA loading plots for the cytokines, demonstrating statistically significant differences when compared to healthy donors. **(A)** cohort infected with original Wuhan SARS-CoV-2 strain (*n* = 59), **(B)** for Alpha variant (*n* = 95), **(C)** for Delta variant (*n* = 78), **(D)** for Omicron variant (*n* = 69), **(E)** for long COVID (LC, *n* = 44).

The loading plots are presented in **Figure 3**.

**Figure 4:**
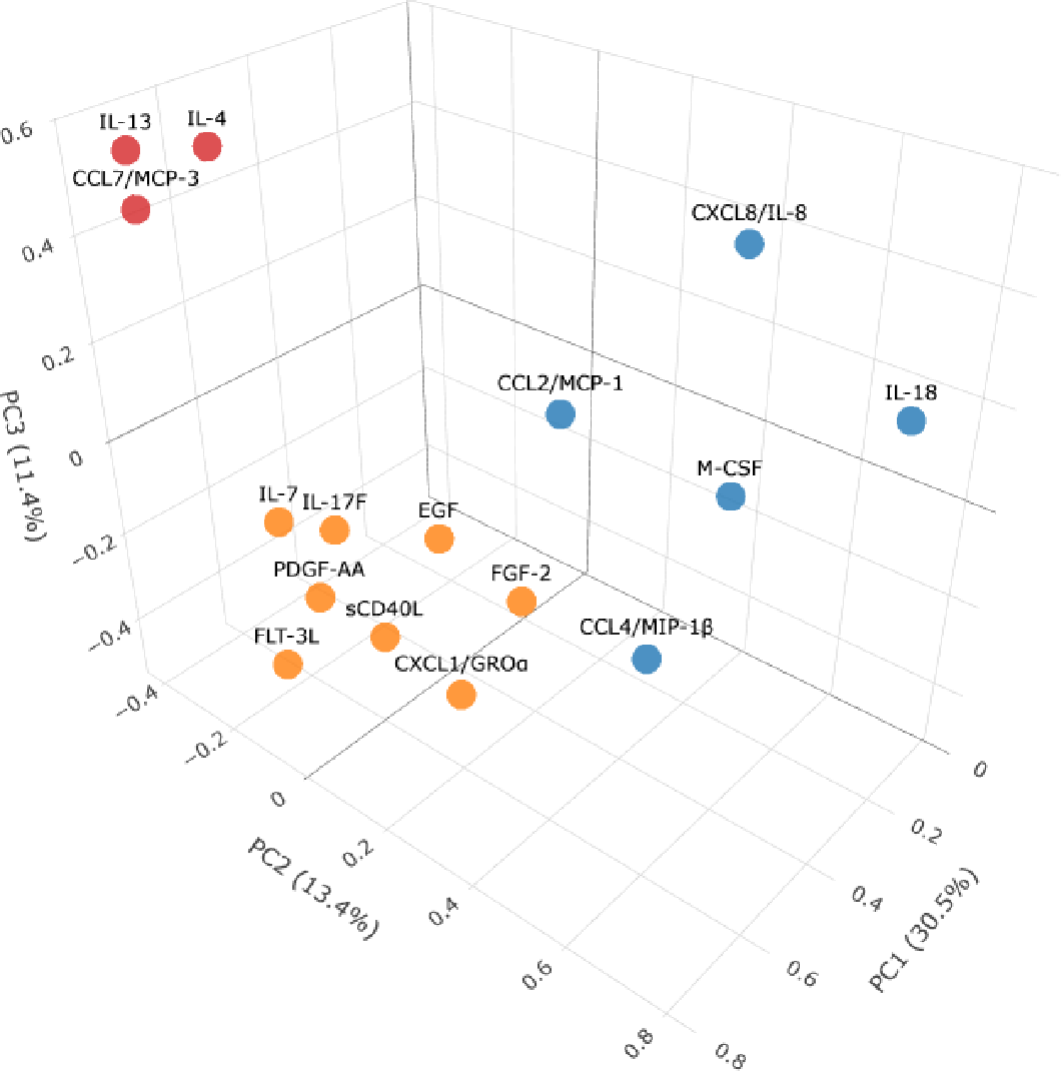
3D clustering of cytokines demonstrating statistically significant differences in LC cohort compared to healthy donors in concentrations based on PCA results. Axes represent PC1–3 and their % of variance. As a result, three key clusters are highlighted: cluster A (red): IL-13, CCL7/MCP-3, IL-4; cluster B (blue): IL-18, CCL2/MCP-1, CCL4/MIP-1β, CXCL8/IL-8, M-CSF, and cluster C (orange): sCD40L, CXCL1/GROα, PDGF-AA, EGF, FGF-2, FLT-3L, IL-7, IL-17F.

For the next step, we performed 3D mapping (**Figure 4**) of key cytokines that demonstrated statistically significant differences and separated them in three clusters based on the results of PCA. Due to insufficient variability across samples (low concentrations close to 0), IL-22 was excluded from further analysis.

## 4 Discussion

### 4.1 Persistent cytokine signature in acute COVID-19 and LC

The cytokines IL-1β, IL-4, IL-6, IL-12 p70, IL-13, IL-15, IL-17A, TNFα, and CCL7/MCP-3 consistently demonstrated importance scores above 0.7 across all cohorts, including the Wuhan, Alpha, Delta, and Omicron variants, as well as in LC patients. The consistent high statistical importance of these mediators across cohorts suggests that their levels are a major source of variation in the immune response to SARS-CoV-2. This makes them strong candidates for playing a key role in COVID-19 pathogenesis, regardless of the viral variant or disease phase.

IL-1β is a significant pro-inflammatory cytokine (15). It is associated with the severe acute phase and persistent symptoms of COVID-19 (16). Their research indicated that IL-1β levels were substantially higher in patients with LC who had been infected by the initial viral variants compared to those infected by later ones, suggesting early strains provoked a more durable peripheral inflammatory response. This aligns with our own study (12), which found that IL-1β was elevated specifically in patients infected with the original Wuhan strain. However, in the LC phase, we observed a decrease in this cytokine. The persistence of LC symptoms despite lower IL-1β levels in later variants suggests the pathophysiology involves more than peripheral inflammation. It may instead involve mechanisms like central neuroinflammation or persistent viral antigens In contrast, the role of the anti-inflammatory Th2 cytokine IL-4 presents a different picture. A separate meta-analysis (17) found that serum IL-4 levels were specifically elevated in non-severe COVID-19 patients compared to healthy individuals, while no significant difference was observed in severe cases. This indicates that a robust IL-4 response may be a characteristic of a more effective, non-severe disease outcome. In a study by Williams et al., IL-4 levels were lower in patients with LC. Our previous study on the subject of Th2-mediated responses in this disease demonstrated no statistical differences between LС and healthy donors (18). Notably, IL-13 demonstrated high importance in the PCA model across all cohorts, even though its plasma levels were not significantly altered compared to healthy donors. This disconnect implies that IL-13’s role, previously linked to COVID-19 severity (19), may be related to its coordinated variation within the immune network rather than its absolute concentration.

IL-6 is one of the highly recognized immune agents for acute COVID-19. The meta-analysis by Jin-Xian Yin et al. (20) provides compelling evidence that elevated serum IL-6 is a significant correlate of LC, with patients demonstrating persistently higher levels compared to healthy controls. This suggests that a state of low-grade systemic inflammation, mediated in part by IL-6, is a hallmark of the condition. However, the failure of IL-6 receptor antagonists like sirukumab to demonstrate a statistically significant clinical benefit in severe and critical COVID-19 patients, as shown in the phase 2 trial by (21), critically questions the simplistic model of IL-6 as a straightforward therapeutic target. This paradox suggests that IL-6’s role in COVID-19 depends not just on its concentration, but also on the dynamics of its soluble receptor (sIL-6R) and other signaling components. The study by Lokau et al. (22) offers a crucial mechanistic insight into this complexity. Their finding that levels of sIL-6R are increased not only in acutely ill patients but also in convalescents after a mild infection reveals a long-lasting alteration of the IL-6 system. Our study, however, demonstrated that IL-6 levels were lower in LC, yet in acute COVID-19 it was one of the most prominent markers of disease-associated changes in the immune system.

Although the pro-inflammatory cytokine TNFα is a well-described component of the COVID-19 cytokine storm and a candidate for therapeutic blockade (23), our data show it was only significantly elevated in Wuhan and Alpha variants. Conversely, LC was linked to low TNF levels. Despite these differences in concentration, TNF retained a high importance score in our PCA model.

IL-12 (also known as IL-12p70) is a critical immunoregulatory cytokine, primarily secreted by antigen-presenting cells. Its expression during an infection is pivotal for directing innate immunity and shaping the subsequent adaptive immune response, notably by stimulating interferon-γ (IFN-γ) production and promoting the differentiation of CD4+ T cells into T helper 1 (Th1) cells. Clinically, research has shown that a combination of elevated IL-10 and IL-12 (p70) levels in patients with two or three comorbidities is a powerful predictor of severe COVID-19, associated with a disease progression risk as high as 97.5% (24). We have observed a decrease in individual IL-12 isoforms, which is consistent with literature data (25). This study also demonstrates a depletion of the pool of other cytokines noted in our work: Il-2, Il-17, interferons and TNFα. This study describes this decrease as a ‘deficiency’ of the cytokine link.

Interleukin-15 (IL-15) is a pleiotropic cytokine that regulates inflammatory and protective immune responses to pathogens by modulating both innate and adaptive immune cells (26). Although its role in COVID-19 is not well characterized, IL-15 has been identified as a predictor of lethal outcome in patients with metabolic syndrome (27). Our analysis reveals a dual aspect of IL-15 in COVID-19: while PCA identified it as a key variable contributing to immune profile variance across all cohorts, its concentration was only significantly elevated in the Wuhan and Alpha variants. This indicates that IL-15 is a consistently influential part of the cytokine network, but its overt dysregulation may be variant-specific.

Interleukin-17A (IL-17A) is a hallmark of the COVID-19 cytokine storm and is linked to worse disease outcomes (28, 29). Interestingly, while we found IL-17A levels to be significantly decreased in LC patients, PCA identified it as a variable of high importance across all cohorts. This suggests that the regulation of IL-17A is a central feature of the COVID-19 immune response, and its significant reduction in LC may itself be a biologically meaningful signature, rather than a lack of involvement.

The chemokine CCL7/MCP-3 also emerged as a key variable with a high importance rate in our PCA. This finding is consistent with its established biological significance, notably from our previous work which identified a 14-fold increase in CCL7/MCP-3 in fatal COVID-19 cases, marking it as a prognostic marker (7). For context, the related chemokine CCL2/MCP-1 is known for recruiting monocytes and memory T cells (30).

Although PCA highlighted numerous analytes with high importance scores, we present only those with statistically significant differences (Mann-Whitney U test, *p* < 0.05) to provide a more targeted and reliable interpretation of the key immune markers distinguishing each cohort.

### 4.2 Cytokine signatures in LC

We focused our interpretation on cytokines that were both statistically significant in group comparisons and had high Importance scores in the PCA. This dual-criterion approach helps identify cytokines that are not only dysregulated but also major drivers of the overall variance in the LC cytokine network.

IL-4 and IL-13 concentrations demonstrated opposite tendencies; while IL-4 was lowered in the blood plasma of LC cohort, IL-13 demonstrated statistically significant increase. Both these cytokines held high Importance rate via PCA. Their complex interactions are often referred to as ‘axis’: while IL-4 drives IgE class switching, IL-13 is a player of tissue remodeling and mucus production (31). Both cytokines are a part of immune responses in autoimmune diseases (32).

Among the cytokines with high Importance rates in the Principal Component Analysis, sCD40L, PDGF-AA, and CCL7/MCP-3 demonstrated a coordinated increase in the blood plasma of the LC cohort. All three are functionally unified by a common cellular source—platelets—and their synergistic role in linking vascular events to inflammatory recruitment. Their coordinated increase may suggest a cascade of potential events: sCD40L acts as a primary inflammatory amplifier upon platelet activation, engaging CD40 on endothelial and immune cells (33). This activation could potentially facilitate the recruitment of monocytes and other leukocytes guided by the chemokine signal of CCL7/MCP-3. This chemokine is implicated in COVID-19 pathogenesis and has been identified as a predictor of disease progression (34).). In parallel, PDGF-AA might contribute tissue repair and remodeling phase, promoting fibroblast proliferation (35). All three mediators suggest a state of persistent platelet activation and aberrant tissue repair processes in the LC cohort.

### 4.3 Three driving clusters of LC

**Figure 5:**
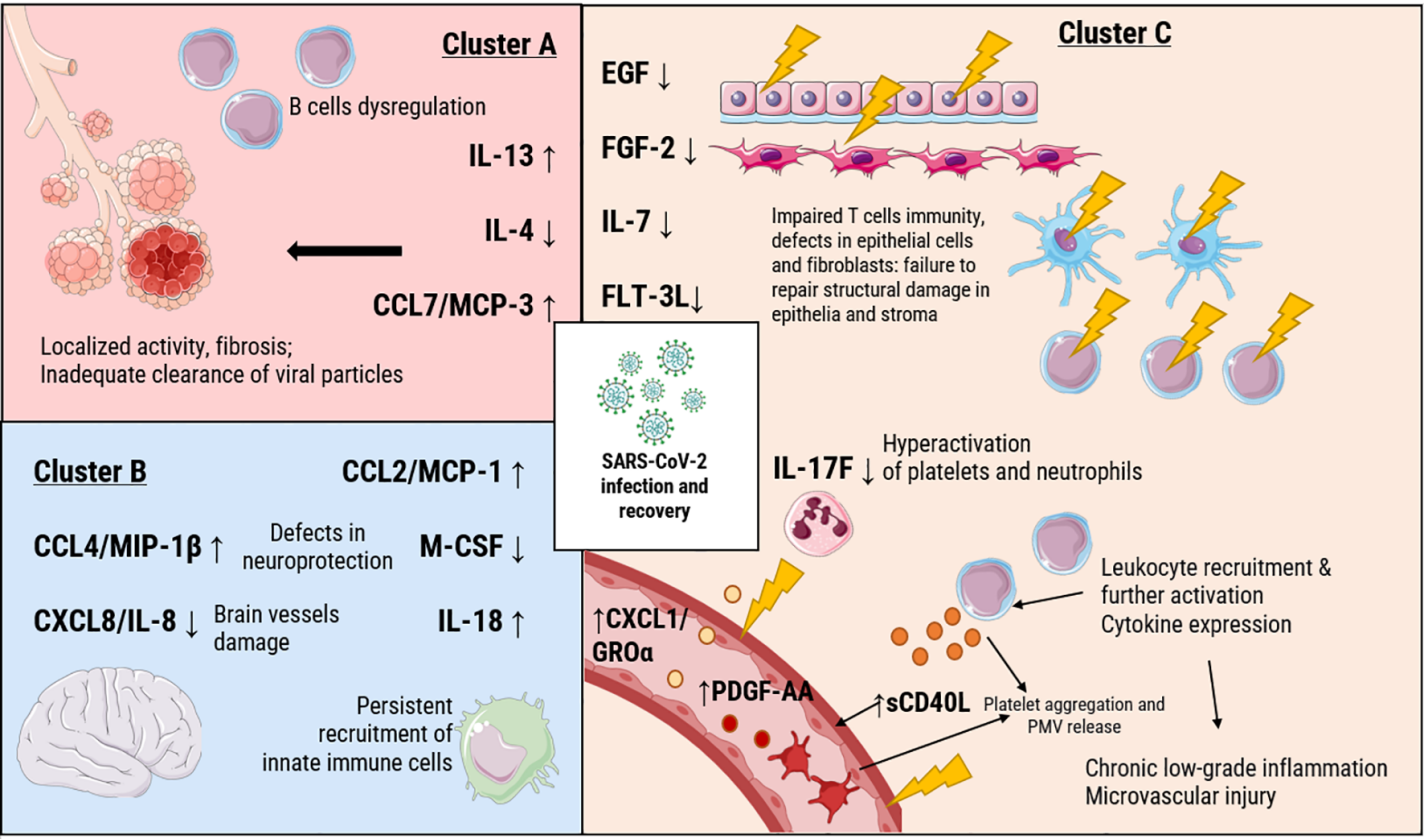
Potential immune pathology behind LC based on the PCA clusterization. Cluster A (red) included IL-13, Il-4, CCL7/MCP-3; cluster B (blue) CCL4/MIP-1β, CXCL8/IL-8, CCL2/MCP-1, M-CSF, IL-18; cluster C (orange): IL-7, IL-17F, EGF, FGF-2, FLT-3L; sCD40L, PDGF-AA, CXCL1/GROα.

Our analysis and 3D visualization identified three principal cytokine clusters associated with LC pathogenesis, as summarized in **Figure 5**.

The cytokines in Cluster A (IL-4, IL-13, CCL7/MCP-3) are consistent with a profile often associated with dysregulated humoral and Th2-mediated immunity. This pattern may indicate a shift in immune responses that could involve B-cells and allergy-like inflammation. The divergent levels of IL-4 (decreased) and IL-13 (increased) point to a dysregulation of the IL-4/IL-13 axis, potentially indicative of a localized, allergy--like inflammatory reaction and bronchial hypersensitivity (36). The inclusion of CCL7/MCP-3, a chemokine responsible for recruiting monocytes, macrophages, and granulocytes (37), points to an insufficient immune response in the airways. This is notable because, while we previously documented an increase in CCL7/MCP-3 in fatal acute COVID-19 cases, its further dynamics in LC indicates a longitudinal immunological shift (7). This chemokine is also involved in vascularization processes (38).

Cluster B, comprising chemokines and IL-18, presents a pattern that aligns with the clinical presentation of LC. It is intriguing to note that some components, like CCL4/MIP-1β and CXCL8/IL-8, have documented roles in processes such as neuroprotection (39, 40). In contrast, other cluster components like CCL2/MCP-1, M-CSF, and IL-18 are potent chemoattractants that recruit innate immune cells (41–43). We hypothesize that the coordinated presence of these factors could represent a mechanism contributing to chronic inflammation, which might also involve the central nervous system.

Cluster C presents the most complex picture, which we interpret as being a dual pathology of immune deficiency and hyperactivation. On one hand, the decreased levels of IL-7 and IL-17F could point toward impaired adaptive immunity and mucosal defense. This may be accompanied by alterations in barrier integrity and dendritic cell function, as suggested by the profile of other cluster members (44, 45). This is compounded by deficits in barrier integrity, affecting both epithelial cells and stromal fibroblasts (46, 47), and disrupted dendritic cell function (48). Elevated levels of sCD40L, PDGF-AA, and CXCL1/GROα point to pronounced activation of platelets and neutrophils. This hyperactive state is strongly associated with the endothelial damage and coagulopathy observed in LC patients and may be a key contributor to these pathologies (49, 50). This is of particular interest, as both COVID-19 and LC involve platelet-mediated pathology.

The pathogenesis of COVID-19 and consequent pathology is significantly driven by platelet-mediated thromboinflammation, with soluble CD40L (sCD40L) serving as a pivotal mediator. As evidenced by Allaoui et al. (51) and Hamzeh-Cognasse et al. (52), platelet-derived sCD40L is markedly elevated in severe infection, directly enhancing platelet aggregation and accelerating clotting, thereby contributing to the characteristic coagulopathy. This prothrombotic state is further by the involvement of other platelet-derived factors. For instance, PDGF-AA, though less studied in this specific context, is known to promote vascular smooth muscle cell proliferation and fibrosis, potentially underpinning the persistent vascular remodeling and endothelial damage observed in LC (53). The inflammatory cascade is amplified by chemokines such as CXCL1/GROα, which contributes to neutrophil-mediated vascular damage by recruiting neutrophils to sites of inflammation or injury via its receptor CXCR2 (54). The synergistic action of sCD40L, PDGF-AA, and CXCL/GROα can lead to a cycle of platelet activation, leukocyte recruitment, and endothelial dysfunction. Furthermore, our findings are consistent with a model in which persistent viral antigens could contribute to sustained platelet activation. Such a mechanism could potentially create a positive feedback loop, perpetuating a pro-thrombotic state that characterizes LC. Impaired phagocytic clearance of SARS-CoV-2 particles may result in their prolonged circulation, providing a continual stimulus for platelet aggregation via direct receptor engagement (e.g., TLRs) or through the formation of platelet-activating immune complexes. This mechanism would create a positive feedback loop, wherein viral persistence perpetuates a pro-thrombotic inflammatory state, contributing to the chronic microvascular injury that characterizes the LC syndrome.

## 5 Conclusions

PCA followed by clustering analysis proved highly effective in identifying not just individual biomarkers, but functionally interconnected cytokine clusters united by common physiological or pathological interactions. Within this study, stable patterns of alterations in the cytokine network associated with COVID-19 and its consequences were identified. Specifically, we noted certain patterns regarding PCA results: IL-1β, IL-4, IL-6, IL-12 p70, IL-13, IL-15, IL-17A, TNFα, and CCL7/MCP-3 was identified by PCA as a major source of variance independently not only of genetic variant of COVID-19, but also in LC. Interestingly, though, for LC most of these molecules were lowered when compared to healthy donors, potentially hinting at certain deficiency or exhaustion.

Our analysis identified three principal immunological clusters underlying pathology behind LC. Cluster A (IL-4, IL-13, CCL7/MCP-3) is indicative of a dysregulated Th2-mediated humoral response. The polarization toward IL-13 implies a localized, allergy-like inflammation, while the deficit in CCL7/MCP-3—a chemokine crucial for myeloid cell recruitment and vascularization—contrasts with its acute-phase elevation and suggests an insufficient airway immune response in the chronic phase. Cluster B (IL-18, CCL2/MCP-1, CCL4/MIP-1β, CXCL8/IL-8, M-CSF) offers insight into innate immune dysregulation, presenting a dual role: components like CCL4/MIP-1β and CXCL8/IL-8 may engage in neuroprotective angiogenesis, while others (CCL2/MCP-1, M-CSF, IL-18) likely perpetuate chronic inflammation and innate cell recruitment, including within the central nervous system. Most complex is Cluster C (sCD40L, CXCL1/GROα, PDGF-AA, EGF, FGF-2, FLT-3L, IL-7, IL-17F), which reflects a paradox of simultaneous deficiency and hyperactivation. It combines markers of impaired T-cell immunity, barrier integrity, and dendritic cell function with elevated factors indicating intense platelet and neutrophil activity, thereby providing a mechanism for the microvascular injury and coagulopathy observed in LC. Further studies are required to assess the state of coagulation factors and blood vessel in this complicated pathology.

The coordinated interactions within these clusters reveal a complex immunopathology behind LC.

## Data Availability

The datasets analyzed for this study are available at the request due to institutional policy on data curation. Requests to access these datasets should be directed to ZK, zoia-korobova@yandex.ru.
